# The Intriguing Life of Autophagosomes

**DOI:** 10.3390/ijms13033618

**Published:** 2012-03-19

**Authors:** Dalibor Mijaljica, Mark Prescott, Rodney J. Devenish

**Affiliations:** Department of Biochemistry and Molecular Biology, School of Biomedical Sciences, Faculty of Medicine, Nursing and Health Sciences, Monash University, Clayton campus, Victoria 3800, Australia; E-Mails: Dalibor.Mijaljica@monash.edu (D.M.); Mark.Prescott@monash.edu (M.P.)

**Keywords:** autophagosome, degradation, lysosome, macroautophagy, mammals, membrane, organelle, yeast

## Abstract

Autophagosomes are double-membrane vesicles characteristic of macroautophagy, a degradative pathway for cytoplasmic material and organelles terminating in the lysosomal or vacuole compartment for mammals and yeast, respectively. This highly dynamic, multi-step process requires significant membrane reorganization events at different stages of the macroautophagic process. Such events include exchange and flow of lipids and proteins between membranes and vesicles (e.g., during initiation and growth of the phagophore), vesicular positioning and trafficking within the cell (e.g., autophagosome location and movement) and fusion of autophagosomes with the boundary membranes of the degradative compartment. Here, we review current knowledge on the contribution of different organelles to the formation of autophagosomes, their trafficking and fate within the cell. We will consider some of the unresolved questions related to the molecular mechanisms that regulate the “life and death” of the autophagosome.

## 1. Autophagy

Autophagy refers to a set of cellular homeostasis processes conserved across all eukaryotes that collectively serve as a tightly regulated intracellular surveillance mechanism, which is indispensable for maintenance of cell health. Induced by cellular stress, such as nutrient limitation, autophagy is the means by which lysosomes in mammalian cells and the vacuole in yeast contribute to the turnover of cellular components (autophagic cargo), including long-lived proteins, macromolecules, whole organelles and even pathogens. By degrading a plethora of (intra)cellular constituents for recycling, autophagy enables cells to survive periods of stress, whether initiated by events intrinsic or extrinsic to the cell. In multi-cellular organisms, impaired autophagic function seems to underlie a range of pathological conditions [1–5].

In mammalian cells, there are three distinct forms of autophagy: macroautophagy, microautophagy and chaperone-mediated autophagy (CMA). CMA is apparently absent from lower eukaryotes, including yeast. The relative contribution of each form of autophagy under different physiological or pathological settings is poorly understood. In CMA, soluble proteins bearing a KFERQ pentapeptide motif are recognized, unfolded and transported directly across the limiting membrane of the lysosome by specific protein machinery [6]. Microautophagy involves the direct engulfment of cargo at the lysosome/vacuole surface by invagination, or protrusion and septation of the lysosome/vacuole membrane [7–9] or internalization by late endosomes (endosomal microautophagy) [10,11]. By contrast, during macroautophagy, cargo is sequestered into a double-membrane vesicle, termed the autophagosome (AP) [12–18]. It is this transient vesicle, the morphological hallmark of macroautophagy, which is the subject of this review. In particular, we will focus on membrane events, including the exchange and flow of lipids and proteins at different stages of the macroautophagic process.

## 2. Macroautophagy

**Figure 1:**
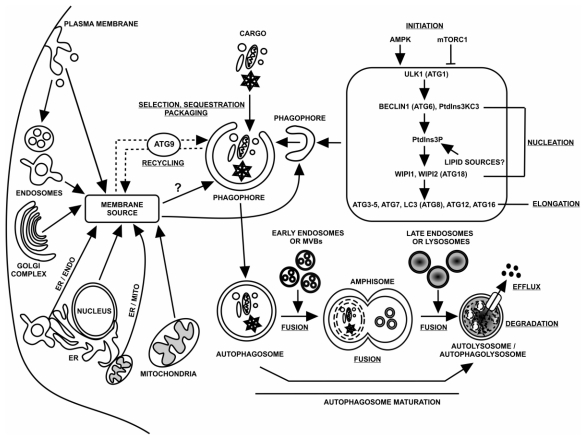
Schematic model of dynamic membrane events contributing to autophagosome life and death. The life and death of autophagosomes, which is evolutionarily conserved, involves the following stages (underlined): initiation, phagophore nucleation, phagophore elongation through two conjugation cascades, cargo selection, sequestration and packaging, membrane recycling regulated by Atg9, fusion with lysosomes, degradation and efflux. Key proteins that act at these stages during such processes are listed. Potential sources for the phagophore include the endoplasmic reticulum (ER), nuclear membranes, mitochondria, plasma membrane, Golgi complex and endosomes. The phagophore expands, sequestering and packaging the selected autophagy cargo, and finally closes, forming an immature autophagosome. During the maturation process, an amphisome is formed from the fusion of an autophagosome with early endosomes or multivesicular bodies, whereas an autolysosome/autophagolysosome is formed as a product of fusion of amphisomes events with late endosomes or lysosomes. Once in the lysosomes, the autophagic cargo is degraded into essential building blocks that are transported back into the cytoplasm. Abbreviations: AMPK, AMP-activated protein kinase; Atg, autophagy-related; BECLIN 1, BCL-2 interacting myosin/moesin-like coiled-coil protein 1; ER, endoplasmic reticulum; ER/ENDO, endoplasmic reticulum/endosomes contact; ER/MITO, endoplasmic reticulum/mitochondria contact; LC3, light chain 3; mTORC1, mammalian target of rapamycin complex 1; MVBs, multivesicular bodies; PM, plasma membrane; PtdIns3P, phosphatidylinositol 3-phosphate; PtdIns3KC3; phosphatidylinositol 3-kinase class III; ULK1, UNC51-like kinase 1; WIPI1/WIPI2, WD repeat domain phosphoinositide-interacting 1/2.

This pathway consists of a number of distinct steps and includes: (1) formation of the phagophore (*i.e.*, the membrane that will become the AP boundary membrane, sometimes referred to as the isolation membrane); (2) phagophore expansion; (3) cargo selection and packaging; (4) AP formation; (5) AP maturation (which may involve AP fusion with endosomes or multivesicular bodies (MVBs)); (6) cargo delivery by fusion of the “mature” AP with the lysosome/vacuole; (7) breakdown of the sequestered cargo to metabolic “building blocks” and (8) efflux of breakdown products [1]. Upon induction of macroautophagy by nutrient deprivation, or some other signal, the formation of the phagophore is initiated. As expansion takes place, membrane curvature is induced to facilitate eventual sequestration of the cargo. A capacity for variable phagophore expansion allows the cell to adjust the size of APs in order to sequester cellular constituents over a very wide range in size, including organelles, such as a mitochondrion [19,20]. Eventually, the two opposing ends of the elongating phagophore membrane fuse to form an AP, thereby sequestering the cargo from the rest of the cell. The outer-membrane of a newly formed AP may then fuse with the boundary membrane of lysosomes (mammalian cells) or the vacuole (yeast cells). Such a fusion event allows the mixing of contents carried by the respective membrane-bound compartments as well as the delivery of the AP sequestered cargo into the acidic lumen (pH ~ 4.5–6). Through the action of resident lysosomal/vacuolar hydrolases, cargo is degraded to basic metabolic building blocks (e.g., nucleotides, amino acids, sugars, fatty acids), which are exported to the cytosol for reuse by the cell (Figure 1) [21].

Macroautophagy utilizes a set of core gene products (many are designated as autophagy-related: Atg), but may require cargo type-specific components also. Although macroautophagy was initially described as a non-selective process in terms of cargo selection, it is now well recognized that particular cell components, structures and organelles can be selectively targeted (Table 1) [22–54].

## 3. Autophagosomes (APs)

As the defining structure of the macroautophagy pathway, the AP is characterized by a number of unique properties: site(s) of initiation, structure, source of membrane lipids, trafficking and fusion events.

### 3.1. Site(s) of Initiation

The first crucial, but least understood, event in AP formation is the induction and nucleation of the phagophore membrane that will grow into the limiting membrane that sequesters cargo. In mammalian cells, multiple sites of AP formation can be detected throughout the cytoplasm [55,56]. In yeast, APs are generated at, or around the pre-autophagosomal structure (PAS), a single functional site situated close to the vacuole membrane [56]. It is not known why the PAS should be located close to the vacuole and how it is actually formed. It has been suggested that it may be physically linked to the ER, which is consistent with the suggestion that it is usually localized at a nuclear-vacuolar junction [14,57,58]. In serving as the phagophore initiation and assembly site the PAS is considered to be a dynamic structure to which many Atg proteins are recruited in an ordered manner and rapidly disassembled from the PAS once they fulfill their purpose [14,57,58].

### 3.2. Structure

The AP can be considerably larger than most other vesicles in the cell and has a membrane that is apparently enriched for a relatively small number of different proteins. This seemingly limited repertoire of proteins suggests that the AP membranes contain only the minimal components necessary to load the cargo destined for eventual degradation and to facilitate subsequent fusion with the limiting membrane of the degradative compartment [59–61]. The compositions of the outer and inner AP membranes seem to be quite different [62], and this may reflect their different roles: cargo sequestration by the inner membrane cargo and fusion with the degradative compartment by the outer membrane. APs in yeast range from 0.4–0.9 μm in diameter [63], whereas mammalian APs are usually larger, being 0.5–1.5 μm in diameter [64–66]. The size of APs may be determined in relation to a specific cargo, which can range from proteins to intracellular bacteria [4]. While the volume of each AP represents less than 0.1% of the total cellular volume in mammalian cells, because the half-life of an AP is considered to be very short (5–10 min), the total degradative capacity of macroautophagy can be large [64–67].

### 3.3. Source of Membrane Lipids

The origin of the AP membranes, whether from pre-existing membranes or formed by *de novo* synthesis, has long been a controversial but fundamental question in the field of autophagy. Recently, a number of studies have suggested the ER, nucleus, mitochondria, plasma membrane, endosomes and Golgi complex may each serve as a source of lipids (Figure 1) [14,15]. The relative contribution of each of these sites to formation of the AP is not presently known. It is possible that different membrane sources are utilized, dependent on the cell type, stress and intended cargo.

### 3.4. Trafficking and Fusion Events

The completed APs loaded with cargo must traffic to and fuse with lysosomes or the vacuole in order to acquire degradative capacity. Moreover, APs can either fuse homotypically with other APs, or receive inputs from the endocytic pathway (Figure 1) by fusing heterotypically with early endosomes or multivesicular bodies (MVBs) to form amphisomes. In turn, amphisomes can fuse with late endosomes. Using time-lapse fluorescence microscopy, Kimura and colleagues [68] have shown that, in mammalian cells, APs do not move far from their site of formation until they are completed. After completion, mammalian APs exhibit rapid vectorial, dynein- and microtubule-dependent movement in the direction of lysosomes; the average velocity of AP movement being 5 μm/s [68]. However, the detailed mechanism by which this process occurs is still far from fully understood.

## 4. Autophagosome Origin and Birth

In this section of the review, we discuss and analyze current understanding of the origin of APs (Figure 1) and the mechanism(s) that lead to AP formation. However, a major challenge in understanding these processes arises from the fact that the different potential membrane sources and mode of transport of lipids from them are only now beginning to be probed rigorously.

### 4.1. ER and Mitochondrial Membranes

Ktistakis and colleagues [69] have reported that in mammalian cells subjected to amino acid starvation, PI3P-enriched structures named omegasomes form in close proximity to ER membranes and Vps34-positive endosomes. An omegasome marker, DFCP1 (a phospholipid binding protein), colocalizes with the autophagy-specific proteins, Atg5 and LC3 (mammalian counterpart of yeast Atg8), which are recruited to sites of AP formation by upstream factors such as the ULK1 complex, the PI3 kinase complex and at a later stage Atg9 (Figure 1), promoting the formation of the curved, cradle-like phagophore by membrane invagination at the centre of the omegasome. Once formed, an autophagic structure seems to exit the omegasome [69,70].

Electron tomography studies [71,72] have delineated the 3D architecture of the relationship between the ER and the phagophore. These studies confirmed that a portion of the ER forms a cradle-like structure surrounding the phagophore such that the phagophore is sandwiched between two ER cisternae. A narrow membrane extension connects the phagophore and ER, giving a rise to the ER-isolation membrane (ER-IM) complex. Immuno-electron microscopy revealed that GFP-DFCP1 localizes to the ER-IM complex, indicating that the cradle is possibly related to the omegasome, at least in that both contain DFCP1 [71,72]. It is possible that the phagophore grows and expands inside the cradle with the associated ER membranes acting as a lipid donor for membrane expansion [14].

Lippincott-Schwartz and colleagues [73] have suggested an alternative model in which DFCP1 may be located at sites where the ER and mitochondria make contact. Rapid formation of APs was proposed to drive lipid transfer from the ER to mitochondria where lipids are modified and then utilized in the formation of APs, which subsequently bud from the outer membrane (OM) of the mitochondrion [73]. The proteins anchored in the outer leaflet of the mitochondrial OM, but not transmembrane proteins of the inner membrane or the matrix proteins, colocalized with the AP markers, Atg5 and LC3. The mitochondrial OM proteins did not label the autophagosomal lumen, but rather appeared in the form of ring-shaped structures. Of possible relevance is the report that phosphatidylethanolamine (PE), which is a membrane component of APs, is produced in mitochondria from phosphatidylserine, which is also abundant in the ER [73–75].

### 4.2. ER and Nuclear Membranes

As the nuclear membrane is continuous with the ER membrane network, the nuclear envelope (inner and outer nuclear membranes) could serve as a source of AP membranes. In this context, a coiling of the nuclear membrane has been observed at locations where viral proteins are enriched during a late phase of *Herpes simplex* type 1 (HSV-1) virus infection of murine macrophages. The formation of LC3-positive AP-like structures then occurs as follows: (1) viral capsids assemble and accumulate in the nucleus of infected cells; (2) during the egress process, HSV-1 capsids fuse with the inner nuclear membrane and acquire their envelope in the perinuclear space; (3) a second step of fusion releases a naked HSV-1 capsid into the cytoplasm; and (4) during this process, some of these capsids are trapped by the emerging 4-layer nuclear-derived APs [76,77]. It is not clear whether viruses other than HSV-1 can promote AP formation by a similar mechanism.

### 4.3. Plasma Membrane

Formation of APs from the plasma membrane (PM) in mammalian cells requires components of the endocytic pathway (Figure 1). Recent data suggest that the PM can contribute to early autophagic precursors, a phenomenon that is dependent on the association of Atg16L1-positive vesicles with the PM through Atg16L1–AP2 (clathrin adaptor protein at the Golgi)/clathrin heavy-chain interactions [78,79]. Subsequent scission of the Atg16L1/clathrin/AP2-associated structures, leading to the formation of early endosomal-like intermediates, is a crucial step that enables the liberation and maturation of these Atg16L1 vesicles into APs. These autophagic precursors subsequently mature to form phagophores and are proposed to represent an earlier stage in AP assembly. The ability of PM to contribute to AP formation in mammalian cells (as described above) may be particularly important during periods of increased autophagy, because the large surface area of the PM may serve as a large membrane reservoir that allows cells periods of AP synthesis at levels many-fold higher than under basal conditions, without compromising other processes [78–80].

### 4.4. Golgi Complex and Endosomes

In yeast, tubulo-vesicular compartments often found adjacent to mitochondria and that originate from the secretory pathway are believed to serve as Atg9 reservoirs. The suggested role of Atg9 reservoirs is to deliver and exchange material, including lipids and possibly proteins with the endocytic system and mitochondria. It is believed that one or more of the Atg9 reservoirs, together with other Atg proteins, act in close proximity to the vacuole as a signal for generation of the PAS and formation of an AP [81–83].

Mammalian Atg9 (mAtg9) is found both on the *trans*-Golgi network and endosomes in nutrient-rich cells, and LC3-positive APs in nutrient-starved cells. It is not known from which compartment mAtg9 traffics to reach the developing AP, but the process does require other Atg proteins, namely, the ULK1 (yeast Atg1 counterpart)/Atg13 complex and Beclin 1 (yeast Atg6 counterpart) as well as lipid kinases to be delivered continuously to the phagophore [84]. Wang and colleagues [85] demonstrate that nutrient starvation induces the tubulation and fragmentation of Atg9-positive Golgi membranes in a manner that is dependent on the membrane curvature-driving protein Bif-1/Endophilin B1 and the class III phosphatidylinositol-3-kinase (PI3KC3) complex II. Starvation-induced Atg9 foci colocalized not only with Bif-1, but also with an early endosome marker, Rab5, and an AP precursor marker, Atg16L. Knockout or knockdown of Bif-1 as well as inhibition of the PI3KC3 complex II, by PI3K inhibitor or knockdown of Beclin 1 or UVRAG, impaired Golgi fission, Atg9 trafficking and LC3 foci formation. Hence, these authors proposed that Bif-1-mediated fragmentation of the Golgi complex during nutrient starvation plays a crucial role in Atg9 trafficking and AP biogenesis in mammalian cells [85].

## 5. Phagophore Expansion and Autophagosome Development

The process of membrane expansion is regulated by a number of different Atg proteins that includes ubiquitin-like (Ubl) proteins, which participate in the two conjugation reactions. Both systems include proteins displaying three different enzymatic activities: ubiquitin-activating enzyme (E1), ubiquitin-conjugating enzyme (E2) and ubiquitin-protein ligase (E3) [56,86]. The first Ubl system essential for autophagy and membrane expansion is responsible for generating an Atg12-Atg5 protein conjugate [56,86,87]. In this system, the *C*-terminal glycine of Atg12 is covalently attached to Atg5 through an internal lysine residue. This process requires the action of Atg7 (E1 enzyme) [88] and Atg10 (E2 enzyme) ([89]. A third protein, Atg16, binds to the Atg5 component of the Atg12-Atg5 conjugate and dimerizes to link a pair of Atg12-Atg5 conjugates. This multi-protein Ubl system is constitutively active and crucial for phagophore expansion and AP formation, but these proteins dissociate from the expanding phagophore before its completion and subsequent fusion with lysosomes [1,90].

A second Ubl system involves the processing and subsequent conjugation of Atg8 (LC3) to PE by sequential action of three Atg proteins: Atg4, Atg7 and Atg3 [1,86,91]. LC3 is proteolytically processed by the protease Atg4, forming an intermediate, cytosolically localized LC3-I. Then, the E1 enzyme, Atg7 activates the processed LC3 and transfers it to Atg3 (functions like E2 enzyme). Atg3 finally conjugates LC3 to PE (lipidation process), resulting in a tight association of LC3-PE to the membrane (membrane-bound LC3-II) where it functions in the formation of the AP [56]. Although LC3-II is inserted into both sides of the phagophore membrane, after fusion of the membrane tips to form an AP, the molecules on the outer face are eventually delipidated by Atg4 and recycled [92]. As an aside, LC3-II levels generally correlate with the number of APs present in the cell, making it the basis for autophagy assays [93].

The two Ubl systems do not act independently. The Atg16-Atg12-Atg5 complex can bring LC3 to the site of lipidation and act as an E3 for LC3-II conjugation [94]. Meanwhile, Atg10, the E2 enzyme in Atg12-Atg5 conjugation, also facilitates the conversion of LC3 to the lipidated form, although LC3 is not a substrate of Atg10 [95,96].

In yeast and mammals, Atg8/LC3 has been found to promote membrane tethering and hemi-fusion, suggesting that it enables the growth and expansion of the forming phagophore/AP *in vitro* [97] and *in vivo* [15,96]. Membrane tethering mediated by LC3-PE leads to membrane hemi-fusion, which is normally a transient intermediate in membrane fusion reactions. Hemi-fusion involves lipid mixing only between the outer and proximal leaflets of the membranes, but not between the inner leaflets [97,98].

Mammalian cells contain a number of Atg8 orthologs that can be divided into two subgroups based on their amino acid sequence homology, where LC3A-C (including two variants of LC3A originating from an alternative splicing event) constitute the LC3 subfamily and GABARAP, GABARAPL1, GATE-16 (also known as GABARAPL2), and GABARAP-L3 constitute the GABARAP/GATE-16 subfamily [99,100]. Of these eight Atg8 orthologs identified in mammals, only LC3B has been extensively studied. LC3B is known to decorate APs and recruit adaptor proteins such as p62 and NBR1 [101,102]. Other Atg8 orthologs such as GATE-16 and GABARAP were initially characterized as intra-cellular trafficking factors [103,104] and later shown to be localized to starvation-induced APs [105]. The occurrence of several Atg8 orthologs in the mammalian system raises the question whether each has a distinct and crucial role in autophagy. Elazar and colleagues [106] have shown that both the LC3 and GABARAP/GATE-16 subfamilies are indispensable for the autophagic process, acting differentially at early stages of AP biogenesis. Thus, the LC3 subfamily is required for elongation of the phagophore membrane, whereas the GABARAP/GATE-16 subfamily is required for a later stage in AP maturation.

## 6. Autophagosome Fusion Events

In yeast cells, APs are formed at the single PAS, adjacent to the vacuole. By contrast, mammalian APs are formed at many sites upon nutrient-deprivation or rapamycin (a pharmacological autophagy inducer) treatment [4]. Furthermore, once completed, APs are transported to endosomes and lysosomes (Figure 1). The mechanisms employed for this directed movement are not well understood. However, it seems that cytoskeletal elements, such as microtubules and actin microfilaments, may play a crucial role. Interestingly, in yeast it seems that neither type of cytoskeletal element is required for bulk autophagy, but that actin microfilaments are essential for selective types of autophagy. In mammalian cells, it has been shown that AP movement and transport to lysosomes depends on microtubules, whereas the role of actin cables in such events remains unclear [107].

In yeast, the AP is transported to the vacuole and the outer membrane of the AP vesicle docks and fuses with the vacuolar membrane, in a process that is dependent upon two proteins, Ccz1 and Mon1, which form a complex that facilitates homotypic vacuole fusion. Other components involved in AP-vacuole fusion are the SNARE proteins Vam3 and Vti1 (found on the vacuolar membrane), Vam7 and Ykt6 (found on the AP), NSF (Sec18), SNAP (Sec17), Sec19, the Rab protein Ypt7, and members of the class C Vps/HOPS complex [21]. After fusion, the AP inner single-membrane vesicle is released into the vacuole lumen and is now termed the autophagic body. Subsequently, the membrane of the autophagic body is broken down and complete degradation of the autophagic body is dependent on resident vacuolar proteases and acidification of the vacuole [21,63,108]. The Atg15 lipase is required for this degradation process [109,110]. Subsequent recycling of the products of controlled degradation occurs via the action of a group of partially redundant vacuolar effluxers, Atg22, Avt3, and Avt4, which mediate the efflux of leucine and other amino acids resulting from autophagic degradation. The release of autophagy-derived and recycled molecules allows the maintenance of cellular metabolism protein synthesis and viability during starvation conditions [111,112].

In mammalian cells, once delivered to lysosomes, APs tether, dock and then fuse with lysosomal membranes in separately regulated events, independent of lysosomal acidification. However, changes in the intracellular lysosomal and AP lipid content (occasioned by metabolic disorders) and/or protein composition may have pronounced effects on the fusion step and thereby affect the overall degradative activity of macroautophagy. Two kinds of fusion can occur between APs and lysosomes: (1) complete fusion that creates a hybrid compartment (the autolysosome/autophagolysosome); and (2) kiss-and-run fusion during which transfer of some content occurs while still maintaining the separateness of the contributing vesicles [4,113–116]. The relative contribution of each form of fusion and whether there is any related physiological significance is currently unclear.

As stated earlier, *en route* to fusion with lysosomes in mammalian cells, APs can fuse with early endosomes or MVBs to form amphisomes (Figure 1). The fusion step involves proteins such as the ESCRT complex, SNAREs, Rab7 and the class C Vps proteins [4,116–118]. Fusion of APs with lysosomes *versus* MVBs may be differentially regulated, as fusion of MVBs with APs is a calcium-dependent event involving Rab11 [119]. UVRAG, a Beclin 1 interacting protein, is involved in the maturation step by recruiting the class C Vps proteins and activating Rab7, which in turn promotes fusion with late endosomes and lysosomes [120]. Furthermore, another Beclin 1 interacting protein, Rubicon, also functions in the maturation of APs. Rubicon is thought to be a part of a distinct Beclin 1 complex containing Vps34, Vps15 and UVRAG that suppresses AP maturation. However, more work is required to definitively characterize the different Beclin 1 complexes and their roles in the autophagy pathway [4,121].

## 7. Outstanding Questions

As described above, APs are responsible for the sequestration of autophagic cargo and its delivery to lysosomes or the vacuole for degradation and recycling. However, a number of questions remain to be addressed.

### 7.1. AP Origin

Although we have improved understanding of the possible sources of lipid for the AP membrane, the relative contribution of each source under any one set of conditions remains to be determined. In this context, what determines whether the membrane comes from any particular source remains unknown. Do the relative numbers of APs formed from any one source change under autophagy-induction or pathological conditions? Do non-selective and selective types of macroautophagy use the same, or different membrane sources for AP formation? How is mobilization of membrane from different sources achieved and how is the supply of various lipids and proteins to the AP membrane regulated?

### 7.2. AP Development

What is the driving force for phagophore curvature and expansion? In addition to known Atg proteins, what are other (co)factors or proteins are necessary for phagophore curvature and AP formation? What are the interactions between these proteins and how such events are regulated? What is the precise molecular mechanism required for sealing of the phagophore membrane to from the AP membrane? What factors regulate AP size and number, and how do these parameters vary under different physiological or pathophysiological conditions? What is the driving force for AP transport/trafficking and how are cytoskeletal elements connected to APs?

### 7.3. AP Maturation and Death

Are different conditions and/or machineries required for APs to undergo fusion with early/late endosomes or MVBs? Are different fusion components required to act on AP membranes having distinct lipid compositions?

Answering these questions will ensure that understanding the “life and death” of autophagosomes remains a vibrant and active area of investigation of both membrane biogenesis and function in the broader context of autophagy for some years to come.

## Figures and Tables

**Table 1:** Selective types of autophagy. For further information on different types of selective autophagy, we refer the reader to the references listed.

Selective type of autophagy	Cargo	Organism	References
Aggrephagy	Protein aggregates	Mammals	[22–25]
Cytoplasm-to-vacuole targeting (Cvt) pathway	Pro-aminopeptidase 1 (prApe1), pro-α mannosidase 1 (prAms1) and aspartyl aminopeptidase (Ape4)	Yeast	[22,26–28]
ER-phagy/reticulophagy	ER	Yeast and Mammals	[22,28–32]
Lipophagy	Lipids	Mammals	[33–35]
Lysophagy/Lysosomophagy	Vacuole/Lysosomal membrane	Yeast and Mammals	[22]
Mitophagy	Mitochondria	Yeast and Mammals	[20,22,36–39]
Nucleophagy	Nucleus	Yeast and Mammals	[22,40,41]
Pexophagy	Peroxisomes/peroxisome cluster	Yeast and Mammals	[22,42–46]
Piecemeal-microautophagy of the nucleus (PMN)	Portions of the nucleus	Yeast	[22,47–49]
Ribophagy	Ribosomes	Yeast	[28,50,51]
Secretophagy	Atg15 protein	Yeast	[52]
Vacuole import and degradation (Vid) pathway	Fructose-1,6-bisphosphatase (FBPase)	Yeast	[22,53]
Xenophagy	Pathogens (bacteria and viruses)	Plants and Mammals	[22,23,54]
